# Long-term costs associated with healthcare use of people with cancer in Scotland

**DOI:** 10.1007/s10198-025-01800-8

**Published:** 2025-06-09

**Authors:** Kenny Haining, Elizabeth Lemmon, Peter Hall, Nazir I. Lone

**Affiliations:** The Usher Institute, The University of Edinburgh, Usher Building, 5-7 Little France Road, Edinburgh BioQuarter - Gate 3, Edinburgh, EH16 4UX Scotland

**Keywords:** Cancer, Costs, Economics, Healthcare use, Healthcare costs, Predictors

## Abstract

**Background:**

Evidence for the long-term costs of cancer is limited, particularly in the Scottish population. Our aim was to better understand the long-term healthcare use and associated costs of cancer in Scotland, and their relationship with cancer survival.

**Methods:**

This was a retrospective study using routine healthcare data to measure inpatient, outpatient, community prescription use and their costs from a national health service perspective. Per-episode incidence costs were assigned using reference costs and charted over eight years during the period 2009 to 2018 by year and phase of care. Risk factors for survival and costs were analysed using Cox regression and generalised linear model regression.

**Results:**

In total, 55,807 adults with cancer were followed over eight years after their diagnosis. Trajectories indicated a complex relationship with survival. Mean cumulative per-patient costs for all cancers were £29,460 at 2017/18 price levels (95% CI £29,199 to £29,720). Considerable variation was observed between cancer types with the highest costs in non-Hodgkin lymphoma at £47,672 (95%CI £45,500 to £49,843) and the lowest in malignant melanoma of skin at £19,217, (95%CI £18,251 to £20,184). Variables negatively associated with costs tended to be positively associated with hazard of death. Only screening was significantly associated with both lower costs (adjusted cost ratio 0.85, *p* < 0.001) and lower hazard of death (adjusted hazard ratio 0.30, *p* < 0.001).

**Conclusions:**

Substantial costs were observed in all cancer types studied, with the highest costs measured in the year following diagnosis. Screening was associated with both lower costs and better survival, supporting the focus on early detection.

**Supplementary Information:**

The online version contains supplementary material available at 10.1007/s10198-025-01800-8.

## Introduction

Changes in risk factors, screening and treatment are improving overall survival after a cancer diagnosis. However, the long-term effects on healthcare use and the associated costs of a cancer diagnosis have received less study than those in the treatment period after diagnosis [1].

Substantial healthcare use and associated costs for people with cancer have been measured in other studies [2–14], but heterogeneity in research questions, methods and populations makes the generalisation of results challenging. Spending on healthcare is considerably higher in the United States (US) than in Europe [15], with cancer drugs costing around twice as much on average [16]. Other European countries may provide closer comparisons to the United Kingdom (UK), however the UK has relatively poor cancer outcomes [17] and its cancer spending of 5% of total healthcare spending is lower than the European average of 6% [18].

Increased healthcare use has been observed in the first year after a cancer diagnosis [19]. Other studies found that healthcare use increased considerably as death approached [2, 20], even when this was up to five years beyond the diagnosis [2] and that the cumulative costs of cancer in subsequent years were comparable to, or even higher than those in the year after diagnosis [2]. Improved survival may increase healthcare use due to ongoing surveillance, recurrences of cancer [21] and the presence of comorbidities [22]. Additionally, there may be long-term impacts from a cancer diagnosis and treatment in survivors, such as cognitive impairment, sexual dysfunction, infertility and premature ageing. Factors associated with high healthcare use include sex, marital status, ethnicity, geographical region [20], and disease stage [5, 19]. Typically, a small proportion of patients typically incur very high costs [23]. Considerable variation in costs has been observed between cancer sites, with prostate cancer found to have lower costs than breast, colorectal [5, 19, 23], and lung cancer [5].

However, evidence in the Scottish population is limited, with studies focused mainly on subgroups of breast cancer using small samples and with few incorporating longer-term costs. Generalising other results to Scotland is complicated by the considerable differences in health within the UK. Scotland’s population has poorer health [24] and lower life expectancy than other western European nations [25–27] due to a complex mix of health behaviours and socioeconomic factors [24, 26, 28]. The mortality rates of lung cancer and heart disease in Scotland are the highest in western Europe [29] while overall cancer mortality rates are higher than in other European nations [25], with a hazard ratio 1.4 times that of England [30].

Scotland’s distinct healthcare system has a high degree of centralisation that offers opportunities to researchers, as routine healthcare datasets can be linked using a unique patient identifier. This analysis utilised the data linkage infrastructure in Scotland to fill the evidence gap on long-term healthcare use and costs as outlined above. Specifically, this study aimed to increase understanding of the economic costs of cancer and their relationship with overall survival, by measuring the long-term healthcare use of people with cancer in Scotland using linked routine healthcare datasets. The objectives were to:

1. Describe the dynamics of healthcare costs over time
2. Examine how costs vary between different cancer types
3. Measure and compare risk factors for costs and overall survival.

## Methods

### Study overview

#### Study design and setting

This was a retrospective cohort study using patient-level data. Multiple routine health datasets were linked to measure the healthcare use of individuals over an eight-year period after a cancer diagnosis. The study population was Scotland’s 14 regional NHS boards, covering approximately 5.5 million people. Eligible patients had a cancer diagnosis recorded in the Scottish NHS Cancer Register, known as Scottish Morbidity Record (SMR) 06 during the period 1 January 2009 to 31 December 2010. Each patient was followed for eight years after diagnosis, with the last possible date of follow-up being 31 December 2018. Additionally, pre-diagnosis healthcare use was obtained for a period of five years before the date of diagnosis. The earliest possible date of the pre-diagnosis period was 1 January 2004, the last possible date 31 December 2010.

A bottom-up gross costing approach was taken, using reference costs from the Scottish Costs Book [31] taking a base-year for costs of 2017/18, to estimate incidence costs over an eight-year period. Cost estimates were presented as cumulative eight-year costs, and were also stratified by year and by phase-of-care.

#### Participants

Inclusion/Eligibility Criteria.

- The patient had an entry recorded in the Scottish Cancer Registry (SMR06)
- The date of diagnosis of the entry occurred during the recruitment period 2009–10
- The patient was alive at diagnosis
- The patient was at least 18 years old.

Exclusion Criteria.

- The patient was under 18 years old at the time of diagnosis
- The patient had a date of death which preceded the cancer diagnosis (indicating an error in linkage)
- The patient’s cancer was not a first cancer, i.e., a previous SMR06 record for the patient prior to the exposure window
- The patient’s cancer was identified during autopsy.

We selected the cohort by filtering the SMR06 dataset according to the above criteria. The full range of ICD10 (version 3) codes included in the cohort was C00–C96 excluding C44.^1^ We stratified the SMR06 cohort by the 10 most common cancers in Scotland, as defined by NHS Scotland incidence rates in 2019 with the ICD10 codes listed below. All SMR06 records belonging to other ICD10 codes were assigned the category *Other malignant neoplasms* and included in the analysis. This gave cancer groups as follows.

1. Trachea, bronchus and lung (C33–C34)
2. Breast (C50)
3. Colorectal (C18–C20)
4. Prostate (C61)
5. Head and neck (C00–C14, C30–C32)
6. Malignant melanoma of skin (C43)
7. Kidney (C64–C65)
8. Non-Hodgkin lymphoma (C82–C86)
9. Oesophagus (C15)
10. Bladder (C67)
11. Other malignant neoplasms

Follow-up was achieved by linking SMR06 records with records in SMR00 (outpatient visits), SMR01 (inpatient episodes) and the Prescribing Information System (PIS) dataset, which records prescribed items dispensed in the community. Linkage was achieved via the Community Health Index (CHI) number, which is unique to each patient. The CHI number was replaced with an anonymised identifier during linkage. If a patient died or moved away during the follow-up period, measurement of healthcare use would continue at a rate of zero for each year after death. The time in days since the cancer diagnosis was calculated as the difference between the admission date and diagnosis date for inpatient records, and the difference between the outpatient visit date and diagnosis date for outpatient visits Data.

Our cohort was derived from the Scottish Cancer Registry as recorded in SMR06. In Scotland, approximately 45,000 cancer registrations are made each year, with more than 1,400,000 recorded since 1958. [33] The Registry records new cases in Scotland of primary malignant neoplasms, carcinoma in situ, neoplasms of uncertain behaviour, and benign brain and spinal cord tumours^2^ [33]. SMR06 does not record episodes, but rather accrued information relating to a primary tumour. This information can include patient data, diagnostic information and treatment, obtained from multiple sources including computerised health records, paper records and external databases such as deaths from the General Register Office [33]. Outcomes data were derived from the SMR00, SMR01 and PIS datasets.

#### Data access, linkage and cleaning

Due to the highly sensitive nature of the data, all dataset linkage was performed by the electronic Data Research and Innovation Service (eDRIS) in a restricted and secure environment. Records were indexed and linked by the CHI identifier, a unique number identifying all patients in NHS Scotland, then identifying information (including the CHI number) was removed and a master index file was created to index anonymised records. All subsequent analyses were performed in a secure environment with outputs thoroughly checked for potentially disclosive information by eDRIS staff before release. Prior to analysis we removed duplicate records and records with a discordant death certificate, where the date of death preceded the diagnosis. For a small proportion (< 0.1%) of SMR06 records the ICD10 code could not be determined, due to duplicate records being recorded on the same day with discordant ICD10 codes. With no way of knowing which code was correct and these records were also removed from the analysis.

#### Data quality

SMR data are regularly audited in accordance with national rules and standards at Scottish Hospitals by the Information Services Division (ISD) Data Quality Assurance team. Previous audits have found that the specialty code was more than 99% accurate for 2010/11 and 2014/15, while the main condition (3-digit) was 88.3% accurate in 2010/11 and 89.0% in 2014/15 [34]. While the existence of NHS Scotland’s CHI number makes novel linkages possible, it has been noted that linkage of data across databases could be improved in Scotland’s health systems [27].

#### Ethical approval and patient consultation

Use of NHS data was approved by the NHS Scotland Public Benefit and Privacy Panel for Health and Social Care (HSC-PBPP) (ref 1819–007). Additionally, the research proposal and lay summary were presented to the Patient-Public Involvement in Research (PPI) group in Edinburgh for feedback. The PPI group was broadly supportive of the project aims, as the topic resonated with some of their experiences during their recovery journey.

#### Cost assignment

Unit costs for inpatient episodes and outpatient visits were assigned using reference costs from the Scottish Costs Book [31]. The assignment of reference costs to hospital episode statistics is believed to be a robust costing method [35]. Episodes were assigned 2017/18 unit costs using the R040 sheet for all study years. Unit costs defined in the Scottish Costs Book for inpatient episodes include resources: medical and dental, nursing, pharmacy, theatre, laboratory, and allied health practitioners (AHPs). Patient Level Information and Costing Systems (PLICS) costs were present in the dataset but only for a small number of years. These were used to calibrate our cost assignment method. We chose per-episode costing as it better approximated the PLICS mean, which is preferred to the median in healthcare costings, and because per-episode costings are less prone to overestimation of costs than per-diem costings [36], which was observed in this data. The use of healthcare resource groups (HRGs) was considered, however reference groups were only available for English NHS costs and these may not be representative of Scottish costs [36]. Costs were reported as pounds sterling at 2017/18 price levels.

## Variables

### Outcome variables

The primary outcome was total costs for an individual, equal to the sum of costs over a defined period of interest for inpatient episodes, outpatient visits and prescriptions. The defined periods of interest were the eight-year post-diagnosis period, individual years of the pre-diagnosis and post-diagnosis follow-ups, and distinct phases of care for cancer survivorship: pre-diagnosis, initial (treatment), continuing, end-of-life. The initial, or treatment, period covered the 12 months after the diagnosis, but could be replaced by the end-of-life period if the patient died within months of diagnosis. The end-of-life period covered the 12 months immediately preceding a patient’s death, or the time from diagnosis to death if this was shorter. If a patient died during the 12 months immediately after diagnosis, all post-diagnosis costs would accrue to the end-of-life period. The continuing period applied to any time between the initial and end-of-life periods. Calculation of total costs required preliminary steps of measuring units of healthcare use and then assigning costs to units of healthcare use. The units of healthcare use were inpatient episodes, outpatient visits and prescribed items.

### Explanatory variables

The explanatory variables age, sex at birth, Scottish Index of Multiple Deprivation (SIMD), method of first detection, rurality, pre-diagnosis costs, NHS Scotland Region Network and disease stage were included in regression models. Also included were 14 comorbid conditions, coded as binary variables, corresponding to components of the Charlson Comorbidity Index.

### Statistical methods

We presented sample characteristics of the cohort in tables stratified by the cancer groupings. Trajectories of cumulative total costs were reported for the eight-year post-diagnosis period. Additionally, we reported trajectories of total costs by phase-of-care. Total costs were the sum of inpatient, outpatient and prescriptions costs. As the objective was to report healthcare use and associated costs for patients after a diagnosis of cancer, rather than cancer-specific costs, all healthcare use was included and cost measurements were unadjusted. The reporting of phase-of-care costs followed recommendations of Wijeysundera et al. (2012) [37]. The initial (treatment) period covered the 12 months after the diagnosis (this could be replaced by the end-of-life period if the patient died within 24 months of diagnosis). The end-of-life period covered the 12 months immediately preceding a patient's death, or the time from diagnosis to death if this was shorter. If a patient died during the 12 months immediately after diagnosis, all post-diagnosis costs would accrue to the end-of-life period. The continuing period applied to any time between the initial and end-of-life periods. Although the initial phase can vary in length for different cancers, we used the same length for all cancers in order to improve comparability.

To identify predictors of costs and survival, univariable and multivariable regression methods were used: Cox regression for survival and generalised linear model (GLM) regressions for costs. Variable selection was based on the cancer literature, expert opinion and availability of variables in the datasets.

Although health costs tend to be dominated by zero values [38], our dataset contained very few,, which may have resulted from the long time frame and also the inclusion of prescriptions, which tend to occur more frequently than hospital visits. Nonetheless, the distribution of health care costs in the cohort remain highly positively skewed and an established approach to deal with this in health economic costing is to use a GLM [38]. The selection of distribution family and log-link function based on sums of squared residuals suggested a gamma distribution with log-link function. Raw coefficients of GLM models can be unintuitive to interpret, therefore we presented coefficients as exponents using Stata’s eform option. This provided an interpretation of a coefficient as a cost ratio (CR), aiding comparison with the hazard ratio in Cox regression. The exponentiated coefficients can be interpreted as simple ratios of baseline costs, with a unit increase in the variable having a cost multiplier of $\beta$. Hence $\beta >1$ increases costs, $\beta <1$ decreases costs, $\beta =1$ leaves costs unchanged. GLM coefficients were estimated by maximum likelihood. Robust standard errors were used throughout. For Cox regressions, residuals and the proportional hazards assumption were analysed visually.

As costs described past expenditure rather than the present value of projected future expenditure, we reported all costs without discounting. The inclusion of yearly cost trajectories allows other researchers to apply custom discount rates. Significance levels of 5% were used and 95% confidence intervals were reported where appropriate. The large sample sizes meant that the confidence intervals for costs were generally narrow and would not be visible in charts of cost trajectories. Hence, they were reported only in tables of results. All analyses were carried out in Stata 15.1.

Where low, moderate and high survival are mentioned we followed the boundaries given by Blakely et al. (2015) [6] with low < 0.25, moderate = 0.25–0.6, high > 0.6. While that study used five-year survival the difference between five-year and eight-year survival in our dataset was minimal and made no practical difference to the cancer sets encompassed.

## Results

### Participants

From a total of 493,882 cancer records for the entire SMR06 database, 55,807 records describing unique participants were included in the final analysis. We excluded 411,367 records that fell outside the study window to give all participants the same length of follow up. An additional 234 records were excluded because their inclusion would have disclosed information about participants. Of the remaining records 26,417 describing second cancers were excluded as these represented patients already included, as well as 57 records with discordant data relating to date of deaths.

### Descriptive data

There was considerable variation in patient characteristics across cancer sites (Table 1). Within the four most common cancers, patients with trachea, bronchus and lung cancers tended to be older, come from more deprived areas, and have more comorbidities than patients with colorectal, prostate cancer and particularly breast cancer. They also tended to have a higher proportion of tumours diagnosed at an advanced stage than patients with breast cancer or colorectal cancer.

**Table 1:** Characteristics of the participants at baseline by cancer type

	Trachea, bronchus and lung	Breast	Colorectal	Prostate	Head and neck	Malignant melanoma of skin	Kidney	Non-Hodgkin lymphoma	Oesophagus	Bladder	All other cancers	All cancers
	*N* = 9,132	*N* = 8,138	*N* = 7,270	*N* = 5,770	*N* = 2,130	*N* = 2,062	*N* = 1,541	*N* = 1,881	*N* = 1,590	*N* = 1,373	*N* = 14,920	*N* = 55,807
Sex												
Male	4718 (51.7%)	46 (0.6%)	3942 (54.2%)	5770 (100.0%)	1481 (69.5%)	905 (43.9%)	877 (56.9%)	932 (49.5%)	1004 (63.1%)	910 (66.3%)	6350 (42.6%)	26,935 (48.3%)
Female	4414 (48.3%)	8092 (99.4%)	3328 (45.8%)	NA	649 (30.5%)	1157 (56.1%)	664 (43.1%)	949 (50.5%)	586 (36.9%)	463 (33.7%)	8570 (57.4%)	28,872 (51.7%)
Age in years (mean, sd)	71.4 (10.6)	62.6 (14.1)	70.4 (11.8)	70.6 (9.4)	63.7 (12.3)	59.5 (17.5)	67.6 (12.9)	66.3 (14.2)	70.2 (11.6)	73.8 (10.9)	66.2 (16.1)	67.5 (13.8)
Age												
< 50	249 (2.7%)	1551 (19.1%)	325 (4.5%)	55 (1.0%)	249 (11.7%)	635 (30.8%)	140 (9.1%)	229 (12.2%)	67 (4.2%)	35 (2.5%)	2348 (15.7%)	5883 (10.5%)
50–59	996 (10.9%)	1896 (23.3%)	952 (13.1%)	599 (10.4%)	500 (23.5%)	355 (17.2%)	261 (16.9%)	316 (16.8%)	226 (14.2%)	103 (7.5%)	2015 (13.5%)	8219 (14.7%)
60–69	2396 (26.2%)	2191 (26.9%)	1909 (26.3%)	2030 (35.2%)	683 (32.1%)	396 (19.2%)	417 (27.1%)	487 (25.9%)	446 (28.1%)	303 (22.1%)	3351 (22.5%)	14,609 (26.2%)
70–79	3334 (36.5%)	1396 (17.2%)	2379 (32.7%)	2026 (35.1%)	479 (22.5%)	400 (19.4%)	428 (27.8%)	504 (26.8%)	481 (30.3%)	463 (33.7%)	3969 (26.6%)	15,859 (28.4%)
> = 80	2157 (23.6%)	1100 (13.5%)	1701 (23.4%)	1059 (18.4%)	219 (10.3%)	276 (13.4%)	295 (19.1%)	345 (18.3%)	370 (23.3%)	469 (34.2%)	3237 (21.7%)	11,228 (20.1%)
Under 65 years old	2247 (24.6%)	4619 (56.8%)	2162 (29.7%)	1588 (27.5%)	1121 (52.6%)	1202 (58.3%)	628 (40.8%)	805 (42.8%)	513 (32.3%)	263 (19.2%)	5951 (39.9%)	21,099 (37.8%)
SIMD quintile												
Least deprived	1005 (11.0%)	1757 (21.6%)	1389 (19.1%)	1306 (22.6%)	247 (11.6%)	559 (27.1%)	274 (17.8%)	392 (20.8%)	234 (14.7%)	238 (17.3%)	2627 (17.6%)	10,028 (18.0%)
2nd least deprived	1339 (14.7%)	1755 (21.6%)	1437 (19.8%)	1273 (22.1%)	350 (16.4%)	448 (21.7%)	274 (17.8%)	368 (19.6%)	289 (18.2%)	248 (18.1%)	2871 (19.2%)	10,652 (19.1%)
3rd least deprived	1734 (19.0%)	1660 (20.4%)	1522 (20.9%)	1238 (21.5%)	409 (19.2%)	435 (21.1%)	331 (21.5%)	404 (21.5%)	337 (21.2%)	303 (22.1%)	3061 (20.5%)	11,434 (20.5%)
2nd most deprived	2256 (24.7%)	1568 (19.3%)	1553 (21.4%)	1089 (18.9%)	488 (22.9%)	338 (16.4%)	343 (22.3%)	377 (20.0%)	355 (22.3%)	288 (21.0%)	3228 (21.6%)	11,883 (21.3%)
Most deprived	2798 (30.6%)	1398 (17.2%)	1369 (18.8%)	864 (15.0%)	636 (29.9%)	282 (13.7%)	319 (20.7%)	340 (18.1%)	375 (23.6%)	296 (21.6%)	3133 (21.0%)	11,810 (21.2%)
Regional network												
South and east	2463 (27.0%)	2168 (26.6%)	2032 (28.0%)	1752 (30.4%)	564 (26.5%)	540 (26.2%)	433 (28.1%)	559 (29.7%)	403 (25.3%)	387 (28.2%)	4101 (27.5%)	15,402 (27.6%)
West	4684 (51.3%)	3820 (46.9%)	3284 (45.2%)	2498 (43.3%)	1089 (51.1%)	997 (48.4%)	715 (46.4%)	818 (43.5%)	761 (47.9%)	603 (43.9%)	7099 (47.6%)	26,368 (47.2%)
North	1985 (21.7%)	2150 (26.4%)	1954 (26.9%)	1520 (26.3%)	477 (22.4%)	525 (25.5%)	393 (25.5%)	504 (26.8%)	426 (26.8%)	383 (27.9%)	3720 (24.9%)	14,037 (25.2%)
Patient recorded as dying *	8638 (94.6%)	2622 (32.2%)	4313 (59.3%)	2570 (44.5%)	1266 (59.4%)	552 (26.8%)	921 (59.8%)	955 (50.8%)	1464 (92.1%)	1024 (74.6%)	10,519 (70.5%)	34,844 (62.4%)
Survival months (mean, sd)	15.9 (24.5)	77.7 (30.8)	54.3 (39.9)	71.2 (33.4)	56.1 (39.1)	81.4 (27.9)	52.9 (40.7)	60.6 (40.2)	19.8 (26.7)	41.4 (38.0)	39.3 (40.7)	49.1 (41.2)
Stage II and below	2793 (30.6%)	6746 (82.9%)	1146 (15.8%)	No info	No info	No info	No info	No info	No info	No info	296 (2.0%)	10,981 (19.7%)
Stage IV	4501 (49.3%)	501 (6.2%)	1276 (17.6%)	No info	No info	No info	No info	No info	No info	No info	222 (1.5%)	6500 (11.6%)
Method of 1 st detection												
Screening examination	NA	2555 (31.4%)	1002 (13.8%)	NA	NA	NA	NA	NA	NA	NA	NA	3557 (6.4%)
Clinical presentation	8606 (94.2%)	5282 (64.9%)	6120 (84.2%)	5513 (95.5%)	2106 (98.9%)	2042 (99.0%)	1325 (86.0%)	1814 (96.4%)	1565 (98.4%)	1340 (97.6%)	13,772 (92.3%)	49,485 (88.7%)
Incidental finding and other	526 (5.8%)	301 (3.7%)	148 (2.0%)	257 (4.5%)	24 (1.1%)	20 (1.0%)	216 (14.0%)	67 (3.6%)	25 (1.6%)	33 (2.4%)	1148 (7.7%)	2765 (5.0%)
Comorbidity count												
Zero	6440 (70.5%)	7415 (91.1%)	6167 (84.8%)	5058 (87.7%)	1796 (84.3%)	1901 (92.2%)	1243 (80.7%)	1565 (83.2%)	1295 (81.4%)	1101 (80.2%)	12,000 (80.4%)	45,981 (82.4%)
One	1996 (21.9%)	577 (7.1%)	853 (11.7%)	547 (9.5%)	253 (11.9%)	125 (6.1%)	218 (14.1%)	239 (12.7%)	237 (14.9%)	201 (14.6%)	2247 (15.1%)	7493 (13.4%)
Two or more	696 (7.6%)	146 (1.8%)	250 (3.4%)	165 (2.9%)	81 (3.8%)	36 (1.7%)	80 (5.2%)	77 (4.1%)	58 (3.6%)	71 (5.2%)	673 (4.5%)	2333 (4.2%)
Comorbidities												
Acute myocardial infarcation	266 (2.9%)	80 (1.0%)	135 (1.9%)	124 (2.1%)	50 (2.3%)	25 (1.2%)	43 (2.8%)	34 (1.8%)	35 (2.2%)	37 (2.7%)	287 (1.9%)	1116 (2.0%)
Congestive heart failure	192 (2.1%)	67 (0.8%)	104 (1.4%)	55 (1.0%)	14 (0.7%)	19 (0.9%)	42 (2.7%)	26 (1.4%)	22 (1.4%)	22 (1.6%)	230 (1.5%)	793 (1.4%)
Peripheral vascular disease	292 (3.2%)	49 (0.6%)	103 (1.4%)	83 (1.4%)	< 30	< 30	31 (2.0%)	< 30	< 30	< 30	186 (1.2%)	867 (1.6%)
Cerebral vascular disease	297 (3.3%)	82 (1.0%)	104 (1.4%)	118 (2.0%)	56 (2.6%)	18 (0.9%)	24 (1.6%)	29 (1.5%)	18 (1.1%)	22 (1.6%)	311 (2.1%)	1079 (1.9%)
Dementia	168 (1.8%)	83 (1.0%)	97 (1.3%)	54 (0.9%)	16 (0.8%)	13 (0.6%)	23 (1.5%)	18 (1.0%)	22 (1.4%)	23 (1.7%)	250 (1.7%)	767 (1.4%)
Chronic pulmonary disease	1404 (15.4%)	226 (2.8%)	279 (3.8%)	172 (3.0%)	116 (5.4%)	48 (2.3%)	60 (3.9%)	74 (3.9%)	92 (5.8%)	79 (5.8%)	676 (4.5%)	3226 (5.8%)
Rheumatoid disease	101 (1.1%)	35 (0.4%)	38 (0.5%)	14 (0.2%)	< 10	< 10	10 (0.6%)	19 (1.0%)	< 10	< 10	103 (0.7%)	349 (0.6%)
Peptic ulcer	40 (0.4%)	24 (0.3%)	33 (0.5%)	11 (0.2%)	12 (0.6%)	< 10	< 10	20 (1.1%)	19 (1.2%)	< 10	159 (1.1%)	334 (0.6%)
Mild liver disease	52 (0.6%)	22 (0.3%)	43 (0.6%)	12 (0.2%)	28 (1.3%)	< 10	16 (1.0%)	20 (1.1%)	19 (1.2%)	< 10	294 (2.0%)	517 (0.9%)
Diabetes	431 (4.7%)	151 (1.9%)	314 (4.3%)	154 (2.7%)	69 (3.2%)	39 (1.9%)	69 (4.5%)	75 (4.0%)	61 (3.8%)	72 (5.2%)	735 (4.9%)	2170 (3.9%)
Diabetes with complications	14 (0.2%)	< 10	< 10	< 10	< 10	< 10	< 10	< 10	< 10	< 10	13 (0.1%)	65 (0.1%)
Hemiplegia	30 (0.3%)	< 10	11 (0.2%)	11 (0.2%)	< 10	< 10	< 10	< 10	< 10	< 10	47 (0.3%)	139 (0.2%)
Renal disease—moderate or severe	209 (2.3%)	63 (0.8%)	112 (1.5%)	89 (1.5%)	12 (0.6%)	10 (0.5%)	56 (3.6%)	45 (2.4%)	23 (1.4%)	55 (4.0%)	317 (2.1%)	991 (1.8%)
Liver disease—moderate or severe	15 (0.2%)	< 10	< 10	< 10	< 10	< 10	< 10	< 10	< 10	< 10	114 (0.8%)	166 (0.3%)

*SIMD* = Scottish Index of Multiple Deprivation; *sd* = standard deviation. Comorbidity measures were recorded prior to SMR06 registration and do not include cancers. Counts are rounded to < 10 and < 30 to prevent disclosure of patient information. * Death may have occurred after the eight-year follow-up

**Fig. 1:**
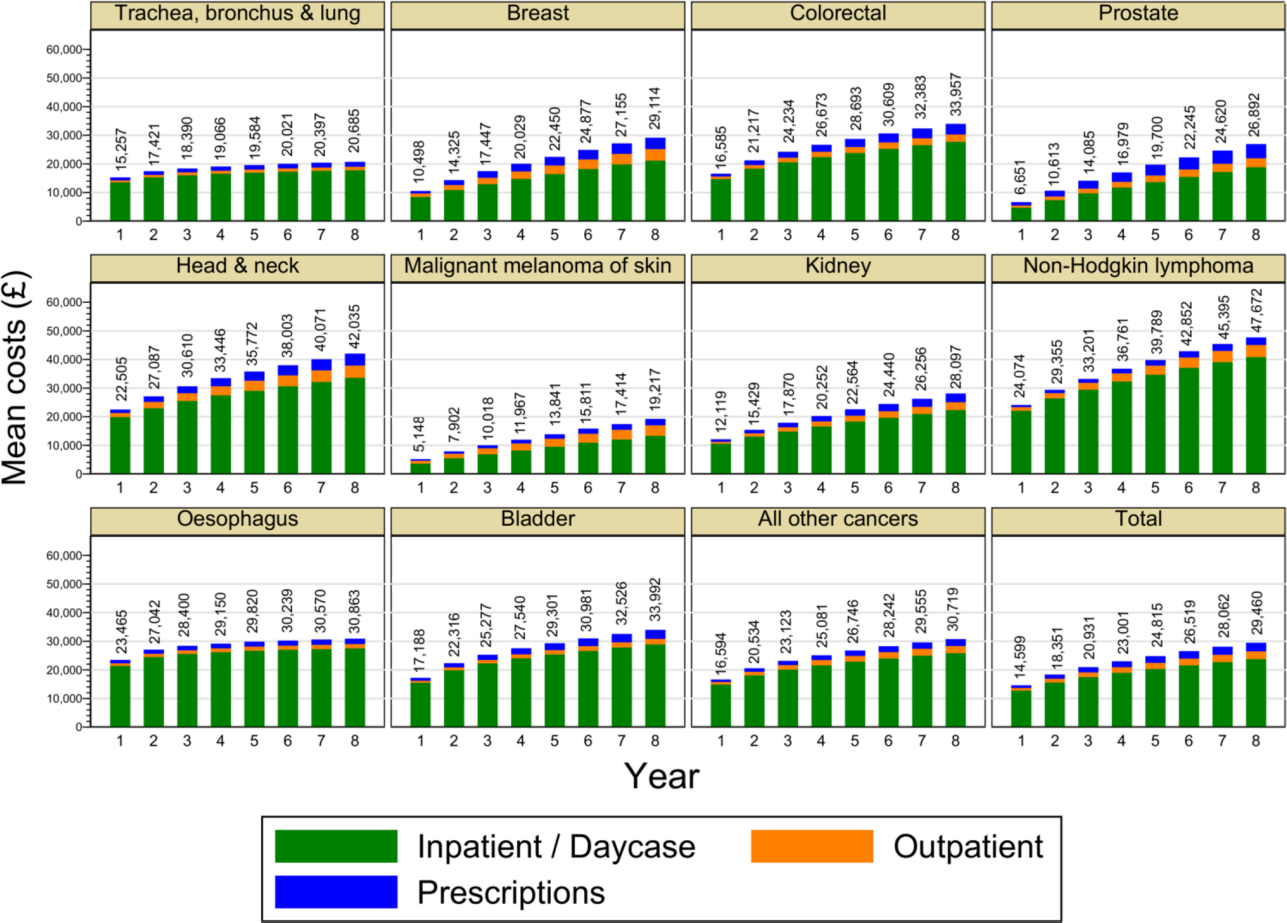
Trajectories of cumulative costs by year and by cancer type, Notes: Years are relative to the diagnosis. All participants were represented in all years. All costs are undiscounted at 2017/18 price levels

**Fig. 2:**
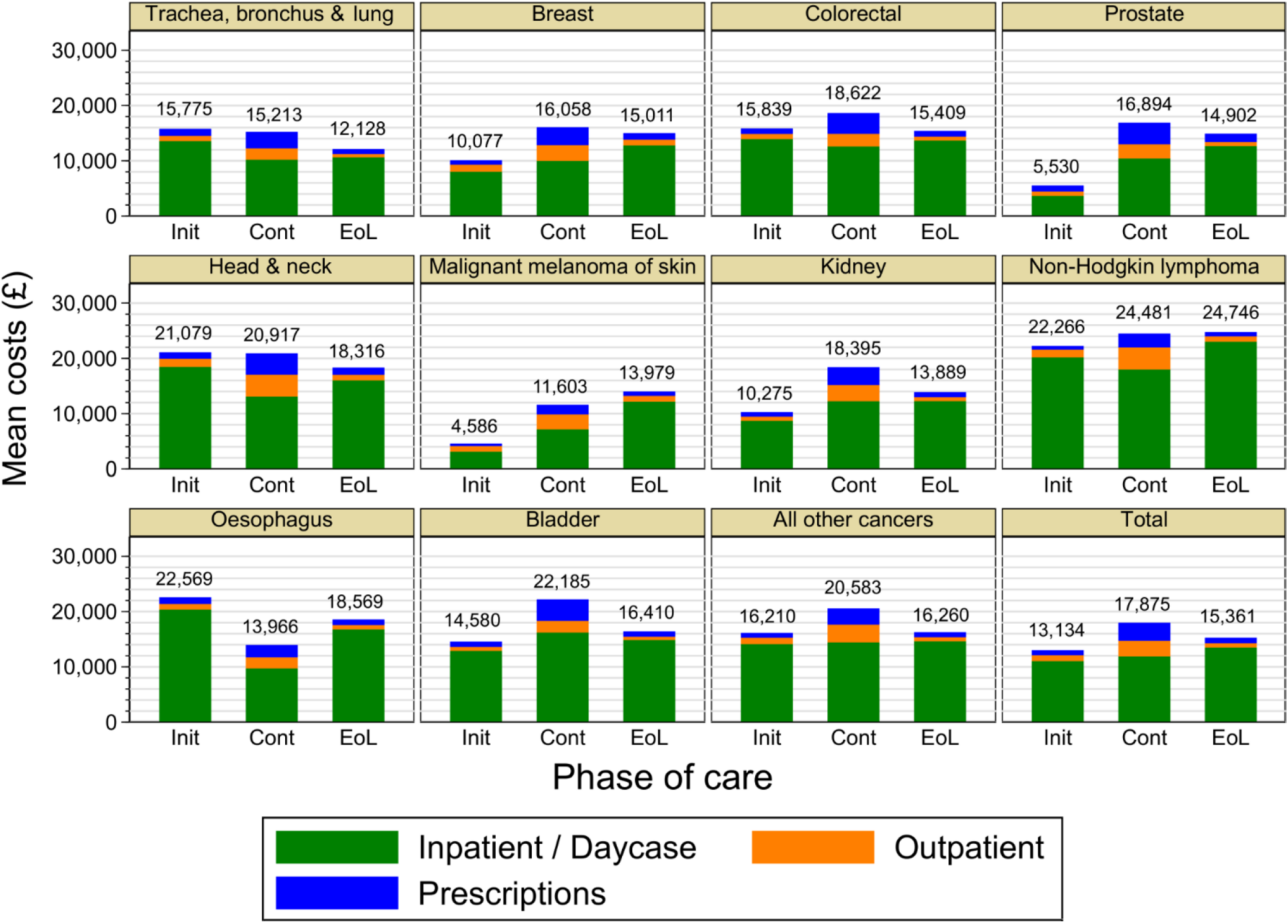
Trajectories of costs by phase-of-care and by cancer type, Notes: Init = initial, Cont = continuing, EoL = end of life. Only participants who entered a phase were represented in that phase. All costs are undiscounted at 2017/18 price levels

Figures 1 and 2 show cumulative costs by year and by phase-of-care and give insights into the relationship between survival and costs. High-mortality cancers had high monthly rates of costs in the initial and end-of-life phases, which contributed to moderately high cumulative costs in year 1. Costs then accumulated more gradually due to lower survival. Cancers with high survival such as skin and prostate had low costs in the initial phase and moderate costs in other phases, leading to low costs in the diagnosis year that then rose steadily throughout the eight-year period, resulting in moderate overall costs. Cancers with moderate survival, such as head and neck cancer, had moderate to high costs in all phases. Year 1 costs were moderately high and thereafter rose steadily, leading to the highest costs. While considerable variation between rates of cost accumulation can be seen in phase-of-care trajectories, all cancers had the highest rate during the end-of-life phase and the lowest rate in the continuing phase. Annual mean costs are shown in Supplementary Table 1 for reference purposes.

Table 2 shows the mean per-patient cumulative costs by cancer site. The highest costs were observed in non-Hodgkin lymphoma at £47,672 (95%CI £45,500 to £49,843) and the lowest in malignant melanoma of skin at £19,217, (95%CI £18,251 to £20,184).

**Table 2:** Eight-year cumulative mean total costs by cancer type and by sex

Cancer type	Mean (£)	95% CI (£)	
All cancers	29,460	29,199	29,720
Trachea, bronchus & lung	20,685	20,263	21,107
Breast	29,114	28,508	29,721
Colorectal	33,957	33,265	34,649
Prostate	26,892	26,299	27,485
Head & neck	42,035	40,555	43,515
Malignant melanoma of skin	19,217	18,251	20,184
Kidney	28,097	26,741	29,454
Non-Hodgkin lymphoma	47,672	45,500	49,843
Oesophagus	30,863	29,596	32,131
Bladder	33,992	32,646	35,339
All other cancers	30,719	30,103	31,334

Costs are the sum of inpatient/daycase, outpatient and prescriptions. Years are relative to the diagnosis. All costs are undiscounted at 2017/18 price levels. *CI* = confidence interval. *NA* = not applicable

### Analysis of risk factors for costs and mortality

Table 3 compares results from GLM models on costs with Cox proportional hazard models on survival. The general pattern of associations was that factors positively associated with mortality were negatively associated with costs, and vice versa. However, there were exceptions. Factors with significant positive associations between both costs and mortality were pre-diagnosis costs and diabetes, while female sex, screening as first method of detection, and rurality had negative associations with both costs and hazard of death, although rurality was only significant at the 5% level in univariable models. Factors negatively associated with costs and positively associated with hazard of death included age, stage IV and comorbidities, most notably dementia. Cox models showed significant associations between SIMD quintile and mortality, with higher deprivation being positively associated with higher mortality. However, there was no clear association between SIMD quintile and costs, with only the second least deprived quintile being significant at the 5% level and only small differences in the magnitude of associations between quintiles.

**Table 3:** Univariable and multivariable results for GLM regression on costs and Cox regression on hazard of death for all cancer patients over the eight-year post-diagnosis period

	Univariable								Multivariable							
	GLM				Cox				GLM				Cox			
Variable	CR	*p*	95% CI		HR	*p*	95% CI		CR	*p*	95% CI		HR	*p*	95% CI	
Age																
< 50	Reference	–			Reference	–			Reference	–			Reference	–		
50–59	0.973	0.157	0.936	1.011	1.820	< 0.001	1.715	1.932	0.998	0.929	0.961	1.037	1.914	< 0.001	1.805	2.031
60–69	0.893	< 0.001	0.862	0.924	2.545	< 0.001	2.411	2.686	0.908	< 0.001	0.877	0.939	2.511	< 0.001	2.379	2.651
70–79	0.774	< 0.001	0.748	0.801	4.237	< 0.001	4.019	4.466	0.778	< 0.001	0.752	0.805	3.706	< 0.001	3.515	3.908
> = 80	0.539	< 0.001	0.519	0.559	7.359	< 0.001	6.981	7.757	0.549	< 0.001	0.529	0.569	5.942	< 0.001	5.630	6.271
Female	0.942	< 0.001	0.925	0.958	0.819	< 0.001	0.802	0.836	0.962	< 0.001	0.945	0.979	0.975	0.022	0.954	0.996
SIMD																
Least deprived	Reference	–			Reference	–			Reference	–			Reference	–		
2nd least deprived	0.963	0.013	0.935	0.992	1.159	< 0.001	1.118	1.201	0.971	0.042	0.944	0.999	1.152	< 0.001	1.111	1.194
3rd least deprived	0.973	0.060	0.946	1.001	1.279	< 0.001	1.234	1.324	0.980	0.139	0.953	1.007	1.220	< 0.001	1.177	1.264
2nd most deprived	0.999	0.923	0.970	1.028	1.470	< 0.001	1.420	1.521	0.999	0.948	0.973	1.026	1.332	< 0.001	1.287	1.378
Most deprived	1.015	0.295	0.987	1.045	1.663	< 0.001	1.608	1.720	0.998	0.889	0.971	1.026	1.505	< 0.001	1.453	1.558
Method of 1 st detection																
Clinical presentation	Reference	–			Reference	–			Reference	–			Reference	–		
Screening examination	0.929	< 0.001	0.899	0.960	0.170	< 0.001	0.158	0.158	0.851	< 0.001	0.820	0.883	0.304	< 0.001	0.282	0.328
Incidental finding and other	0.886	< 0.001	0.847	0.926	1.005	0.840	0.958	1.054	0.907	< 0.001	0.870	0.946	0.965	0.138	0.919	1.012
Pre-diagnosis costs (per £1000)	1.003	< 0.001	1.000	1.000	1.017	< 0.001	1.000	1.000	1.006	< 0.001	1.000	1.000	1.009	< 0.001	1.000	1.000
Rural	0.983	0.071	0.965	1.001	0.877	< 0.001	0.858	0.896	0.992	0.430	0.974	1.011	0.954	< 0.001	0.931	0.978
Region Network																
West	Reference	–			Reference	–			Reference	–			Reference	–		
South and east	1.001	0.949	0.979	1.023	0.916	< 0.001	0.894	0.939	1.021	0.057	0.999	1.043	0.958	0.002	0.933	0.984
North	0.903	< 0.001	0.885	0.923	0.948	< 0.001	0.924	0.973	0.932	< 0.001	0.912	0.952	0.982	0.215	0.955	1.010
Stage																
II or below	0.995	< 0.001	0.976	1.015	0.433	< 0.001	0.420	0.446	0.992	0.487	0.971	1.014	0.608	< 0.001	0.590	0.627
IV	0.687	< 0.001	0.668	0.706	3.575	< 0.001	3.481	3.670	0.691	< 0.001	0.673	0.710	3.136	< 0.001	3.041	3.234
Acute myocardial infarction	0.959	0.269	0.891	1.033	1.743	< 0.001	1.639	1.854	1.038	0.281	0.970	1.110	1.077	0.035	1.005	1.154
Congestive heart failure	0.679	< 0.001	0.626	0.735	2.588	< 0.001	2.420	2.768	0.795	< 0.001	0.735	0.860	1.334	< 0.001	1.225	1.454
Peripheral vascular disease	0.843	< 0.001	0.789	0.900	2.166	< 0.001	2.033	2.307	0.911	0.003	0.856	0.969	1.272	< 0.001	1.181	1.369
Cerebral vascular disease	0.699	< 0.001	0.657	0.745	2.190	< 0.001	2.056	2.333	0.811	< 0.001	0.761	0.864	1.263	< 0.001	1.169	1.365
Dementia	0.381	< 0.001	0.354	0.409	3.496	< 0.001	3.290	3.714	0.513	< 0.001	0.476	0.552	1.687	< 0.001	1.547	1.840
Chronic pulmonary disease	0.862	< 0.001	0.829	0.897	2.119	< 0.001	2.040	2.201	0.912	< 0.001	0.879	0.947	1.416	< 0.001	1.357	1.477
Rheumatoid disease—Connective tissue	1.027	0.596	0.931	1.134	1.643	< 0.001	1.471	1.835	1.055	0.260	0.961	1.159	1.205	0.002	1.070	1.356
Peptic ulcer	1.027	0.619	0.925	1.141	1.640	< 0.001	1.466	1.836	1.036	0.480	0.940	1.142	1.294	< 0.001	1.142	1.466
Mild liver disease	0.978	0.636	0.890	1.074	1.905	< 0.001	1.728	2.099	0.892	0.013	0.814	0.976	1.770	< 0.001	1.577	1.986
Diabetes	1.044	0.043	1.001	1.089	1.761	< 0.001	1.684	1.840	1.103	< 0.001	1.059	1.148	1.206	< 0.001	1.148	1.267
Diabetes with complications	1.240	0.100	0.960	1.603	1.717	< 0.001	1.382	2.132	1.220	0.136	0.939	1.585	1.208	0.214	0.897	1.628
Hemiplegia	0.960	0.651	0.804	1.146	2.105	< 0.001	1.784	2.484	0.998	0.983	0.854	1.167	1.497	< 0.001	1.230	1.822
Moderate or severe renal disease	0.945	0.245	0.860	1.039	2.401	< 0.001	2.259	2.552	1.037	0.366	0.958	1.124	1.348	< 0.001	1.256	1.446
Moderate or severe liver disease	0.926	0.389	0.777	1.103	2.116	< 0.001	1.778	2.520	0.828	0.041	0.692	0.992	1.508	< 0.001	1.214	1.874

Abbreviations: *GLM* = generalised linear model; *CR* = cost ratio; *HR* = hazard ratio; *SIMD* = Scottish Index of Multiple Deprivation

## Discussion

### Key results

Healthcare use and associated costs were substantial after a diagnosis of cancer, with markedly higher costs in the year after diagnosis than in other years. Considerable variation between cancers was observed. The highest eight-year costs were found in non-Hodgkin lymphoma and the lowest in malignant melanoma of skin. The highest rates of cost accrual were observed in the initial and end-of-life phases, but cumulative costs over the eight-year follow-up were highest in the continuing phase. A complex relationship between survival and costs was observed across cancer types. Malignant melanoma of skin had the highest survival rate but also the lowest cumulative costs, while cancers with very low survival such as trachea, bronchus and lung had lower cumulative costs than cancers with higher survival such as non-Hodgkin lymphoma. Factors positively associated with higher costs tended to be negatively associated with mortality. Exceptions were pre-diagnosis costs and diabetes, both having positive associations with costs and mortality, and screening as method of first detection, which was negatively associated with both costs and mortality.

### Interpretation

In general, factors positively associated with hazard of death tended to be negatively associated with costs, reflecting the importance of survival on long-term costs. An exception was that diabetes was associated with both higher costs and higher mortality. The association with stage was non-linear, with the highest costs tending to be associated with mid-stage cancers. As different cancers tend to be detected at different stages this could explain some of the variation between cancer types. Why higher costs were observed in mid-stage cancers could be a result of the probability of curative treatment being given and the treatment’s aggressiveness. Patients with early-stage cancers are more likely to survive longer, but will require less aggressive treatments. Patients with late-stage cancers are more likely to die sooner and less likely to undergo curative treatment, with given treatments likely to be less aggressive, which would result in lower costs.

Heterogeneity across cancer types was observed. However, the reference costs used were not cancer-specific, meaning that the costs of treatments and drugs for specific cancers were aggregated across all cancers. As lengths of inpatient stays were also not accounted for, it is possible that differences in costs between cancer types were underestimated.

In all phases of care, inpatient costs contributed more to total costs than did outpatient and prescription costs combined. However, their contribution was lower in the continuing period, reflecting lower hospital stays and higher outpatient visits and prescription costs. Prescription costs were comparable to outpatient costs and became relatively more important over the long-term, making a substantial contribution to overall costs in the continuing period. Monthly costs were highest in the end-of-life phase and lowest in the continuing phase for all cancers. However, the cumulative effect over the entire follow-up meant that for all cancers but lung cancer and oesophagus cancer, the continuing phase had higher costs than the initial phase. Additionally, for all cancers except malignant skin melanoma, non-Hodgkin lymphoma and oesophagus cancer, the continuing phase had higher costs than the end-of-life phase.

Inpatient costs have been recorded as the largest proportion of direct costs in other studies [39]. This analysis found that inpatient costs remained the largest component of costs throughout an eight-year follow-up. However, the method of calculating inpatient costs can substantially affect their magnitude. Furthermore, when costs are measured over a longer period, discrepancies between costing methods may be amplified by the presence of longer stays. While costings based on HRGs may be more accurate, per-episode approaches are considered to be of acceptable accuracy [36]. As the foci of our analysis were the dynamics of costs and differences between cancers, rather than precise costing, per-episode costings gave a reasonable compromise between precision and complexity.

### Comparison with other studies

Banegas et al. (2018) [5], using a phase-of-care approach for total and net costs, found lung cancer more expensive than breast, colorectal and prostate cancers, in contrast to our findings. However, breast and colorectal were found to have similar costs and prostate lower, in agreement with our measurements. Costs overall were considerably higher than our measurements—converted to sterling—but this study measured a US population of patients on a health plan, with costing methods geared to the US private healthcare system. Higher drug costs in the US may partly explain the higher costs overall, and population differences may also have contributed. This may also have been the case with Yabroff et al. (2008) [7] who like us studied a wide range of common cancers and found costs highest in the initial and end-of-life phases, with survival and stage as major cost factors. Other areas of agreement were the high costs incurred for non-Hodgkin lymphoma, head and neck cancer and oesophagus cancer, and much lower costs for skin cancer and prostate cancer. However, as with Banegas et al. (2018) [5] and unlike our results, lung-related cancers were found to have relatively high costs.

Studies of UK populations are likely to provide the most relevant comparisons to our analysis. Laudicella et al. (2016) [2] calculated nine-year incidence costs for NHS England cancer survivors, with results more similar in magnitude to our findings than those of Banegas et al. (2018) [5] and Yabroff et al. (2008) [7]. However, the method measured costs only for patients alive at the start of each follow-up period, with the means in each period summed to provide nine-year cumulative costs. While this method is likely to be informative for costs accruing to survivors, it contains a form of survivor bias that is likely to overestimate societal costs; by considering only costs for cancer survivors, it ignores the economic costs accruing to mortality, as the counterfactual—the costs that would have occurred if the patient had not died of cancer—is unmeasured. Marti et al. (2015) [23] also found breast and colorectal more expensive than prostate and that a small number of patients incurred very high costs, however, the small sample size, different population and focus on societal costs, including out-of-pocket costs, in addition to the shorter follow-up period may make results less comparable. Hall et al. (2015) [19] using HRG and PLICS costings to measure 15-month cumulative costs for patients in England, also found breast and prostate to have similar costs that were notably higher than prostate, and that stage was a strong predictor of costs. We found higher costs in the continuing phase than other studies, which was likely due to the longer time frame of our study and the inclusion of community prescription costs.

### Strengths and limitations

Patient-level data covering the Scottish population provided a large and varied enough sample to describe healthcare use for the 10 most common cancers and other cancers combined. Other studies have tended to focus on four common cancers: lung, breast, colorectal and prostate. An eight-year follow-up provided information on how healthcare use and associated costs developed over time. Additionally, the use of PLICS data for calibrating our costing method enhances confidence in our results.

There were also limitations. Public administration data are believed to be a weak source of information on comorbidities [40] and the SMR data have known limitations in accuracy [34]. Common conditions are under-recorded in SMR01 [34] meaning that confounding effects in regression models may not be fully accounted for which could bias the coefficients of other explanatory variables. An issue with outpatient data was that in previous years only new consultations were compulsory to report for some health boards, which may have led to under-reporting of outpatient visits. This was likely to have caused underestimation of costs, however the low contribution of outpatient costs makes it unlikely this was substantial. Data on people who moved abroad were not available in our dataset. As these individuals would still be counted in the follow-up and would accrue zero costs after emigrating (even if they used healthcare abroad), costs would have been underestimated. Additionally, around 8.5% of the Scottish population use some form of private healthcare [41] which may also have exerted downward bias on cost estimates due to non-capture of healthcare use data.

People with cancer have been reported to accrue higher expenditure relating to mental health [42]. The inclusion of SMR04 records, which relate to mental health, would have given a more complete picture of healthcare expenditure. The possibility of accessing this dataset was examined but the additional ethical and privacy issues burdens were considered too high. Due to the limited number of variables captured, this analysis does not purport to be a full description of healthcare costs.

### Generalisability

The health of Scotland’s population is known to differ from other European nations [24], and its geography and healthcare system are also unique, hence generalisability may be limited. Even within the UK there are differences that may limit the external validity of results. Prescriptions are free for patients in Scotland, which may increase demand relative to countries where patients must pay the full price or a contribution to the price, as in England. However, prescription costs were a relatively small component of costs. The remoteness of much of Scotland should be considered, as remote communities have been found to incur higher health costs [43]. It should also be remembered that changing factors, such as new therapies, limit the predictive value of all costing studies.

### Policy implications and future research

People with the four most common cancers in Scotland (lung, breast, colorectal, prostate) accounted for under half of all the spending on healthcare measured in this analysis. However, some less common cancers, most notably non-Hodgkin lymphoma, saw higher per-person spending. Combined, the spending on patients with less common cancers was higher than the four most common cancers combined. This, and the considerable cost variation between cancers, suggest notable cost reductions could be found by targeting less common cancers with high costs such as non-Hodgkin lymphoma and head and neck cancer.

While a complex relationship between survival and costs across cancer types was observed, at the patient level, factors associated with higher survival tended to be associated with higher costs. If cancer survival improves, policymakers should be prepared for increased healthcare costs.

It should be noted that stage at diagnosis is not directly comparable between tumour types and this makes between-tumour cost comparisons difficult to interpret. Further research might usefully focus on sub-populations of cancer such as “treated with curative vs palliative intent”, particularly when informing policy on early diagnosis which might down-shift stage and increase the proportion of cancers that might be curable.

The rates of cost accumulation were highest in the treatment and end-of-life phases but over the long-term costs in the continuing phase were the most substantial in magnitude for patients who entered this phase. The contribution of prescriptions became more pronounced during the continuing phase and was of similar magnitude to outpatient visits. This suggests that the omission of prescriptions from the long-term costs of disease may bias the costs downwards, but also suggests an area for further investigation where costs might be reduced.

## Conclusion

In this analysis we linked routine NHS datasets to measure patient-level healthcare use and associated costs of people with cancer over eight years in the Scottish population. The large sample size allowed us to break down results by the 10 most common cancers and other cancers combined. Incorporating prescriptions provided a fuller account of healthcare costs over the long term. This will improve the understanding of how costs evolve over time and their relationship with survival.

## Supplementary Information

Below is the link to the electronic supplementary material.

Supplementary file1 (XLSX 11 KB)

## Data Availability

Due to their sensitive nature, the data are not freely available. Researchers seeking further information or access to the data should contact eDRIS.

## Footnotes

1 C44 is not recorded in SMR06 because non-melanoma skin cancers (NMSC) are excluded from analyses of all cancers to enable comparison with other countries that do not record NMSC data, and because only the first occurrence of the most common type of NMSC is collected due to high incidence [32]

2 From the year 2000 onward.

## Declarations

This project was funded by the Medical Research Council (MRC). We have no other interests to declare. We would like to acknowledge the electronic Data Research and Innovation Service (eDRIS) team (Public Health Scotland) for their support in obtaining approvals, the provisioning and linking of data and facilitating access to the National Safe Haven. Finally, we would like to acknowledge that this work uses data provided by patients and collected by the NHS as part of their care and support.
